# Decomposing Income-Related Inequalities in Self-Reported Depression and Self-Rated Health Among Married Immigrants in South Korea

**DOI:** 10.3390/ijerph16101869

**Published:** 2019-05-27

**Authors:** Jihyung Hong, Jaehee Lee

**Affiliations:** Department of Healthcare Management, College of Social Science, Gachon University, Seongnam 13120, Korea; jihyung.hong.kr@gmail.com

**Keywords:** income-related inequality, concentration index, depression, self-rated health, immigrant

## Abstract

Health inequalities among immigrant minorities have been under-researched in South Korea. This study, therefore, measured the extent of income-related inequalities in self-reported depression and self-rated health (SRH) among married immigrants in South Korea and decomposed them into sociodemographic determinants using data from the 2015 National Survey of Multicultural Families (*n* = 15,231). The mean age of this sample was 37.8 years (SD = 10.8) and the mean duration of residence was 10.1 years (SD = 7.4). Eighty-five percent were female, and of these, 86.5% were from low/middle-income countries. Of these married immigrants, 34.6% reported experiences of depressive symptoms in the past year, and 9.5% reported their current health to be poor or very poor (weighted). The results also indicated substantial pro-rich health inequalities with the Erreygers concentration index of −0.1298 for self-reported depression and that of −0.1231 for poor SRH. Socioeconomic positions, reflected in income, subjective social status, and employment status, alongside satisfaction with a spouse, appeared to have much greater contributions to the overall inequality than demographics and type of migration. These findings suggest that social welfare policies and programmes can play important roles in reducing health inequalities that are ‘avoidable and unnecessary’ among married immigrants in South Korea.

## 1. Introduction

International migration, often economically motivated, has been increasingly common across the globe over the past decades. The number of international migrants worldwide has reached 258 million in 2017, up from 173 million in 2000 [1]. A similar trend has also been observed in South Korea, which has long been ethnically and culturally homogenous. The number of foreigners/immigrants has rapidly increased over the past two decades, rising from 308,339 (48.0% for long-term residents) in 1998 to 2,180,498 (72.6%) in 2017 [2]. While this rapid increase has been highly driven by work-related immigration (composed mainly of non-professional foreign workers and overseas Korean workers), marriage immigration has also made a considerable contribution. For instance, marriage immigrants accounted only for 1.6% of all marriages in 1993 but 7.9% in 2017 [3].

In South Korea, the rise of international marriage since the early 1990s has been primarily led by brokered international marriage, which is often referred to as ‘mail-order marriage’ [4]. This type of marriage was originally initiated by local governments and civil organisations in order to resolve ‘bride shortages’ in rural areas, with the so-called ‘Matching Drive for Rural Bachelors’ campaign [5]. The Korean Wind phenomenon, a massive return-migration of ethnic Koreans in China beginning in the early 1990s, made these campaigns possible. International marriage brokers have then facilitated this type of marriage, especially in the mid-2000s, leading to a shift from ‘coethnic international marriages’ to ‘interethnic international marriages’, with women mostly from China, and more recently from Southeast Asian countries such as Vietnam and the Philippines [5,6].

Migration can be associated with a number of stress factors that can affect health and well-being, including financial strains, employment difficulties, acculturation challenges, discrimination, lack of social support, and language barriers [7]. According to the 2015 national survey on multicultural families conducted in South Korea, about one in three married immigrants/nationalised citizens were found to have experienced depressive symptoms in the past year [8], which is about triple that reported among native South Koreans [9]. Existing evidence suggests that socioeconomic disadvantage plays a major role in ethnic health inequalities [10,11]. That is, those ethnic minorities/immigrants in better socioeconomic positions generally have better health, implying no inherent link between being an ethnic minority and health [12]. While there are not many studies that have explicitly measured the extent of socioeconomic health inequality within and/or across ethnic minorities/immigrants, recent studies have provided some evidence of income-related health inequality, favouring the rich, among these individuals. For instance, a recent Chinese study, using the concentration index approach, found considerable pro-rich inequality in self-reported health among immigrant workers in China [13]. A study by Mangalore and Knapp also reported similar findings with mental health across various ethnicities in the UK [12]. The role of income on immigrants is, however, not clear in South Korea, as shown in a recent review [6]. The review identified key risk factors that influenced mental health of married immigrant women in South Korea. These included acculturative stress, country of origin, family stress, lack of social support, low level of marriage satisfaction, domestic violence, and extended family structure, but not income. Income was not identified as a key risk factor because of its mixed results in the literature. The authors postulated that financial strain, not an actual level of income, could more consistently influence mental health of married immigrant women in South Korea. It should, however, be noted that many of the reviewed studies focused on small subgroups of married immigrant women. It is, therefore, not clear as to whether the samples were sufficiently representative of the immigrant population across various levels of income.

This study, therefore, aimed to examine and confirm whether and to what extent income-related inequalities exist in self-reported depression and self-rated health (SRH) among married immigrants in South Korea, using the 2015 National Survey of Multicultural Families, the largest data on multicultural families in the country. The concentration index approach was employed to quantify the extent of income-related health inequalities. This study also decomposed these concentration indices into their determinants, which can help draw policy implications. Particular attention was paid to the type of migration, which could differ by sex and country of origin by level of development (low/middle-income countries vs. high-income countries). It would be of interest from a policy perspective whether the type of migration influences the income-related health inequality, independently of other socio-economic factors.

## 2. Materials and Methods

### 2.1. Data and Study Sample

This study used data from the 2015 National Survey of Multicultural Families, which is a cross-sectional survey implemented every 3 years starting from 2009. The survey focuses on marriage immigrants/nationalised citizens and their family members to collect detailed information on household demographics, socio-economic status, social relationship, health status, and social well-being. The 2015 survey aimed to include about 10% (*n* = 27,120 households) of the total population, and of these, 65.8% (*n* = 17,849 households) completed the survey. The present study included married immigrants (*n* = 15,321) from 92 countries (*n* = 3290 from China; *n* = 2635 from Vietnam; *n* = 2108 from China (Korean Chinese); *n* = 1426 from Japan; *n* = 1358 from the Philippines; and *n* = 4504 from the rest), who were still married (i.e., living with their spouses) and whose original nationality was not South Korean. 

This study used secondary data that had been made publicly available after de-identification. A request for an institutional review board (IRB) exemption was, therefore, submitted to the IRB of Gachon University, and the board confirmed this study to be eligible for exemption from IRB review. 

### 2.2. Variables

#### 2.2.1. Dependent Health Variables

Self-reported depression was measured using the following question: ‘During the last 12 months, have you ever felt sadness or despair for more than two weeks, which severely interfered with your daily lives?’. The following four options were provided to the respondents: never (1), occasionally (2), frequently (3), and very frequently (4). Given the ordinal nature of this variable, which is not appropriate for the concentration index approach, it was categorised into two groups: no self-reported depression (1) and self-reported depression (2–4). 

Another health outcome, SRH, was also measured using the following question, as used by the World Health Organisation [14]: ‘How would you rate your overall health?’. The following five options were provided to the respondents: very good (1), good (2), moderate (3), bad (4), and very bad (5). Similarly, this variable was also categorised into two groups: poor SRH (4–5) and no poor SRH (1–3).

While both depression and SRH were assessed with single-item measures, their validity and reliability have been confirmed in the literature [15,16,17,18].

#### 2.2.2. Demographic and Socio-Economic Variables

The following demographic and socioeconomic variables were included in this analysis: age, sex, years of residence in South Korea, area of residence (rural vs. urban), disability card holder (yes vs. no), level of satisfaction with a spouse measured with a five-point scale (very dissatisfied, dissatisfied, fair, satisfied, very satisfied), perceived discrimination measured with a modified version of the Experience of Discrimination questionnaire, which asks the respondents whether they have ever experienced discrimination (yes vs. no) and other further details on the places of discrimination experienced [19], no one around for help when they were sick (yes vs. no) as a proxy for social relationship, level of education (≤middle school, high school, ≥university), employment status (regular work, temporary work, not working), level of average monthly gross household income in the past year (<1 million KRW, 1–2 million KRW, 2–3 million KRW, 3–4 million KRW, 4–5 million KRW, 5–6 million KRW, 6–7 million KRW, ≥7 million KRW (purchasing power parities: 1 U.S. $ = 866 KRW [20], exchange rate: 1 U.S. $ = 1130 KRW [21])), and subjective social status measured with a three-point scale (top, middle, bottom). 

Given that type of married migration is likely to differ by sex and home country by level of development, this study also included the product of these two variables. Home countries were divided into low/middle-income countries and high-income countries, as defined by the World Bank [22]. A substantial proportion of women from low/middle-income countries were likely to have entered South Korea through brokered international marriage, especially in the mid-2000s, whereas a high proportion of men from low/middle-income countries could have entered the country for economic opportunities prior to marriage and have pushed into low-quality jobs (i.e., dirty, dangerous, and demeaning jobs). While the process of migration was likely to differ between women and men from low/middle-income countries, economic pressures could, nonetheless, be the one that triggered both types of migration. 

### 2.3. Statistical Analysis

#### 2.3.1. Concentration Index

The concentration index (CI) approach [23,24] was employed to measure the extent of income-related inequalities in the prevalence of self-reported depression and poor SRH. Similar to Lorenz curves, concentration curves can be plotted with the cumulative percentage of a health outcome (i.e., depression or poor SRH) on the vertical axis corresponding to the rank of income on the horizontal axis. The CI is defined as twice the area between the concentration curve and the 45° line, which ranges from a minimum value of −1 to a maximum of +1. The values of −1 and +1 occur when the health outcome is concentrated in the very poorest or very richest, respectively. A zero value indicates complete equality in the prevalence of the health outcome regardless of income level. The CI can be computed easily by making use of the ‘convenient covariance’ as shown below:

(1) $$CI=\;\frac{2}{\bar{y}}\;COV\left(y_{i},\;R_{i}\right)$$

where $y_{i}$ is the health variable, $\bar{y}$ is the mean of $y_{i}$, and *R_i_* is the fractional rank of the *i*th individual and cov(.) denotes the covariance. 

Health variables may be correlated to age and sex, both of which could possibly be unequally distributed across income groups. This study, therefore, calculated age- and sex-standardised CI to control for the confounding impact of demographic variables. Health variables were first standardised by age and sex using the indirect standardisation method as follows (${\hat{y}}_{i}^{IS}$) [25]:

(2) $${\hat{y}}_{i}^{IS}=y_{i}-{\hat{y}}_{i}^{X}+\bar{y}$$

where $y_{i}$ is the actual value of the health variable, ${\hat{y}}_{i}^{X}$ is the predicted value of the health variable after running a probit model with age and sex exploratory variables (X) controlling for income and other socioeconomic factors included in the decomposition analysis, and $\bar{y}$ is the mean of the health variable. This standardised health variable, which was used to calculate age- and sex-standardised CI, can be interpreted as the distribution of health that would be expected to be observed, irrespective of how age and sex is distributed across the socioeconomic factors. 

#### 2.3.2. Erreygers CI: Corrected Concentration Index

The use of binary variables, however, complicates the measurement of inequality [26,27,28]. For instance, the CI will vary with the mean level of the health variable [26], making its comparison across different populations or health outcomes problematic. The value of the CI will, therefore, vary on whether the focus of the analysis is the occurrence of health outcome or no occurrence of health outcome. That is, the use of the standard CI does not satisfy the mirror condition for bounded variables [26,27]. In addition, the range of the CI will not lie between −1 and +1, but lie between $\bar{y}-1$ and $1-\bar{y}$ (in large samples) [28]. Both Erreygers and Wagstaff hence proposed corrected versions of the CI to adjust for these issues. Erreygers CI can be calculated using the following formula:

(3) $$E_{c}=\;\frac{4\bar{y}}{y^{max}-y^{min}}CI$$

where $y^{max}-y^{min}$ is the range of the health variable, which is ‘one’ in the case of binary variables. 

Wagstaff CI can also be calculated simply by diving the standard CI by ($1-\bar{y}$) in the case of binary health variables. Given that both corrected CIs are commonly used in the health literature, the present study focused on the Erreygers CI but also examined the Wagstaff CI in sensitivity analyses. 

#### 2.3.3. Decomposition of the Erreygers CI

This study also conducted a decomposition analysis, using the method proposed by Wagstaff et al. [29], to examine what and how many demographic and socio-economic factors, respectively, contribute to the overall income-related health inequality. Wagstaff et al. showed that for any health variables exhibiting a linear relationship with a set of k exploratory variables, the CI for the health variable can be decomposed as follows:

(4) $$CI=\;\underset{k}{\sum }\left(\frac{\beta_{k}{\bar{x}}_{k}}{\bar{y}}\right)CI_{k}\;+\frac{GCI_{\varepsilon }}{\overset{-}{y}}$$

where $\beta_{k}$ is the partial effect, $\bar{y}$ is the mean of the health variable, ${\bar{x}}_{k}$ is the mean of $x_{k}$, $CI_{k}$ denotes the concentration index of $x_{k}$ against income, and $GC_{\varepsilon }$ is the generalised concentration for the error term. Notably, the partial effect (dy/d$x_{k}$) of each variable was estimated at the mean of other variables in a probit model. For categorical variables, which were included as a series of dummy variables in the model (i.e., *n*−1 dummy variables for the categorical variable having *n* categories), the ‘margins’ command was used to set ‘other dummy variables belonging to the same categorical variable’ to zero in STATA SE/10 (StataCorp LCC, College Station, TX, USA) [30]. The above formula can be modified as shown below to decompose the Erreygers CI [31]:

(5) $$E_{c}=\;4\left[\underset{k}{\sum }\left(\beta_{k}{\bar{x}}_{k}\right)CI_{k}\;+GCI_{\varepsilon }\right]$$

All analyses were weighted to take into account the sampling strategy. All CIs were calculated using the ‘conindex’ command [32] in STATA SE/10 [30]. 

## 3. Results

### 3.1. Sample Characteristics and Prevalence of Self-Reported Depression/Poor SRH

This analysis included a total of 15,231 married immigrants. The mean age of this sample was 37.8 years (SD = 10.8) and 85.0% (*n* = 13,022) were female (unweighted). The mean duration of residence was 10.1 years (SD = 7.4). Of women, 86.5% were from low/middle-income countries, such as China (*n* = 4518 including Korean-Chinese) and Vietnam (*n* = 2619). More than half of men (60.8%) were also from low/middle-income countries such as China (*n* = 880 including Korean-Chinese) and Pakistan (*n* = 143). Of these married immigrants, 35.1% (*n* = 5382) reported an experience of depressive symptoms in the past year, and 8.1% (*n* = 1237) reported their current health to be poor or very poor (weighted prevalence: 34.6% and 9.5%, respectively). 

Table 1 describes the (weighted) prevalence of self-reported depression and poor SRH, respectively, by sociodemographic factors. The prevalence of depression was highest among married immigrant women from low/middle-income countries (37.0%), followed by married immigrant women from high-income countries (31.7%). The prevalence was, on average, lower among married immigrant men, especially those from high-income countries (23.0%) (*p* < 0.001). Meanwhile, the prevalence of poor SRH was highest among married immigrant men from low/middle-income countries (14.5%) but, again, lowest among married immigrant men from high-income countries (5.0%) (*p* < 0.001). Satisfaction with a spouse appeared to have significant health implications, depression in particular, among married immigrants. About 70.9% of those married immigrants very dissatisfied with their spouse reported experience of depressive symptoms in the past 1 year, whereas only 22.5%—albeit still high—of those very satisfied with their spouse reported experience of depressive symptoms in the same period (*p* < 0.001). In addition, the prevalence of depression was generally higher among those who were aged 65 years or older, disability card holders, less educated, temporary/day labour workers and/or not working, or among those who had experienced discrimination, had no one around for help when they were sick, or perceived their social status to be low. Similar patterns were also observed for the prevalence of poor SRH, but with greater disparities by demographics.

### 3.2. Income-Related Inequality in the Prevalence of Self-Reported Depression and Poor SRH

**Figure 1:**
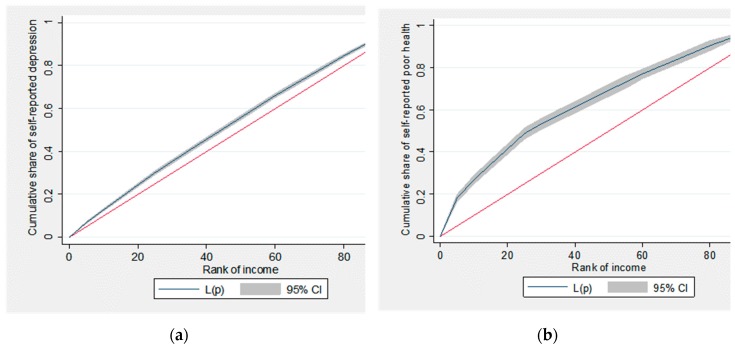
Concentration curves for self-reported depression and poor SRH. (**a**) Concentration curve for self-reported depression; and (**b**) concentration curve for poor SRH. Abbreviations: SRH, self-rated health.

The prevalence of self-reported depression and poor SRH also varied significantly with income level (Table 2), which is directly or indirectly related to many of the above socio-demographic factors. Figure 1 displays the concentration curves for income-related depression and poor SRH, respectively, among these married immigrants in South Korea. Both concentration curves laid above the 45° equality line, implying that both self-reported depression and poor SRH were more concentrated among lower income groups. The curve for poor SRH was even further away from the equality line, meaning greater pro-rich inequality in this outcome. These translated into the CI of −0.0937 (SE = 0.0070) for self-reported depression and that of −0.3247 (SE = 0.0189) for poor SRH (Table 2). This pro-rich inequality was still maintained even when age and sex were standardised, although it became smaller. While Erreygers CI, which adjusted for the binary nature of these health outcomes, also suggested pro-rich inequality in self-reported depression and poor SRH, these estimates turned out to be similar for both health outcomes (−0.1298 (SE = 0.0097) for self-reported depression and −0.1231 (SE = 0.0072) for poor SRH). 

### 3.3. Decomposition of the Erreygers CI into Contributing Factors

The Erreygers CI was decomposed into its determinants to examine how much of the measured inequality was due to income itself and how much was due to other demographic and socioeconomic factors. The results of this decomposition analysis are shown in Table 3. The column ‘CI’ represents the distribution of the determinant itself across income ranks and, therefore, they are the same across different types of health outcomes. The CIs show that individuals who had longer duration of residence, experience of discrimination, higher level of educational attainment (i.e., at least university degree), higher level of income, higher level of subjective social status, or were very satisfied with their spouses were more concentrated in the upper tail of income distribution. On the other hand, those people who were older, female, rural residents, disability card holders, not working, or had no one around for help when they were sick were more concentrated in the lower tail of income distribution. 

The marginal effects from the probit analysis (β) can refer to a point change in the probability of a dependent health variable associated with a unit change in each determinant. The results indicate that longer duration of residence, being middle-aged, living in rural areas, higher satisfaction with spouses, higher income, and higher subjective social status were associated with a lower probability of depression. Meanwhile, having disabilities, having experience of discrimination, having no one around for help when they were sick, and having no permanent employment (i.e., temporary employment or not working) were associated with higher probability of depression. Similar findings were also observed for poor SRH, but with some exceptions. Firstly, being older and the duration of residence were associated with higher probability of poor SRH. Secondly, while the level of education was not statistically significantly associated with the probability of depression, it was statistically significantly associated with lower probability of poor SRH. Similarly, the product of sex and country of origin by level of development, as a proxy for type of migration, was not statistically significantly associated with the probability of depression, but it was so with poor SRH. Compared to men from high-income countries, the rest had a higher probability of poor SRH. 

**Figure 2:**
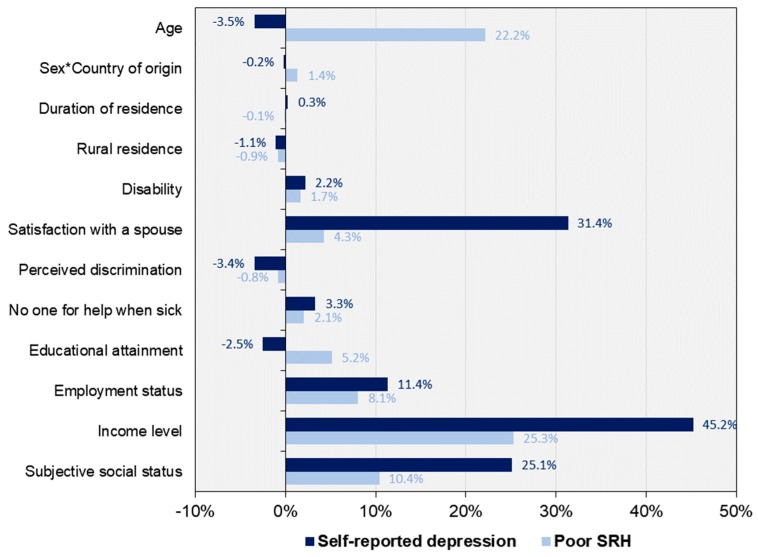
Contribution (%) of each determinant to the total income-related inequality in self-reported depression and poor SRH. Note: This graph describes contribution (%) of each socio-demographic determinant to the concentration index for self-reported depression and poor SRH, respectively. Abbreviations: SRH, self-rated health.

The column (Cont.), next to the marginal effects, shows the absolute contribution of each determinant to the overall income-related health inequality (i.e., Erreygers CI). A determinant would have a greater contribution if it is more unequally distributed across income ranks (i.e., CI), a unit change in the determinant is associated with a greater change in the probability of health outcomes (i.e., β), or if it has a higher mean ($\bar{X}$). Of those socio-demographic determinants, income itself had the largest contribution to the overall income-related inequality both in self-reported depression and poor SRH. The contribution of income, as a share of the overall inequality (i.e., Erreygers CI), was 45.2% for self-reported depression and 25.3% for poor SRH (see Figure 2). The second largest contributor was, however, different between the two health outcomes. While satisfaction with a spouse accounted for 31.4% of the overall inequality in self-reported depression, it explained only 4.3% of the overall inequality in poor SRH. Instead, the contribution of age took up 22.2% of the overall inequality in poor SRH. Of the rest, subjective social status and employment status also made considerable contribution to the overall inequality in both outcomes. Subjective social status accounted for 25.1% and 10.4% of the overall inequality in self-reported depression and poor SRH, respectively. Similarly, employment status explained 11.4% and 8.1% of the overall inequality in self-reported depression and poor SRH, respectively. Notably, the product of sex and country of origin by level of development had little impact on the overall income-related inequality in both outcomes (−0.2% for depression and 1.4% for poor SRH). 

## 4. Discussion

This study assessed the extent of income-related inequality in self-reported depression and poor SRH among married immigrants in South Korea, and decomposed it into its determinants, using data from the 2015 National Survey of Multicultural Families. Of these married immigrants, 34.6% reported experience of depressive symptoms in the past year, and 9.5% reported their current health to be poor or very poor. Women from low/middle-income countries exhibited the highest prevalence of self-reported depression, whereas men from low/middle-income countries revealed the highest prevalence of poor SRH. Notably, men from high-income countries appeared to be at the lowest risk of both outcomes. The results indicated substantial pro-rich health inequalities with the Erreygers CI of −0.1298 for self-reported depression and −0.1231 for poor SRH. The decomposition analysis confirmed that type of migration, as measured with the product of sex and country of origin classified by level of development, had little contribution to the overall income-related health inequality. Instead, socioeconomic factors, such as income, subjective social status, and employment status, played important roles in the overall inequality. Satisfaction with a spouse also made considerable contribution to the overall inequality in self-reported depression. Taken together, these findings suggest that social protection policies and programmes can play important roles in reducing inequalities in self-reported depression and poor SRH that are ‘avoidable and unnecessary’ among married immigrants in South Korea. 

### 4.1. Income-Related Inequality in the Prevalence of Self-Reported Depression and Poor SRH among Married Immigrants

This study revealed the high prevalence of self-reported depression (34.6%) among married immigrants, especially women from low/middle-income countries (37.0%). These estimates appeared to be much higher than that reported among native South Koreans. According to the 2017 Korea National Health and Nutrition Examination Survey, the prevalence of self-reported depression among native South Koreans was 11.2% (9.1% for men and 13.4% for women) [9], which was only one-third of the present estimate for married immigrants. The prevalence of poor SRH, however, appeared to be lower among married immigrants (9.5%) than among native South Koreans (17.8% in 2016) [33], which could be due to age and healthy immigrant effects. That is, married immigrants are, on average, younger and healthier than native South Koreans, especially upon arrival. This is probably because it is easier for young and healthy individuals to decide to migrate and to successfully manage migration (e.g., passing rigorous health screening) [7]. Nevertheless, existing evidence suggests that the healthy immigrant effect tends to diminish over a period of time [7,34], possibly due to acculturation stress and socioeconomic disadvantages. This could be partly reflected in our prevalence estimates. For instance, the prevalence of poor SRH was 14.5% among men from low/middle-income countries but only 5.0% among those men from high-income countries, although the former could have gone through more rigorous immigration controls, including health screening, implying the level of their health at least similar to that of the latter upon arrival. 

The findings also confirmed substantial pro-rich inequality both in self-reported depression and poor SRH among married immigrants in South Korea. This is consistent with those reported in other studies with immigrants and/or ethnic minorities, although very few available with the concentration index approach. For instance, Shao et al., using data from the 2012 China Labour-force Dynamics survey, reported the CI of −0.0866 in SRH among migrant workers in China [13]. Similarly, Mangalore and Knapp, using data from a nationally representative survey of ethnic minorities, measured income-related inequality in mental health problems with income ranks assigned within each ethnicity, as well as across all types of ethnicities in the UK [12]. The study reported that lower income had more significant health implications among African Caribbean, Pakistani, or Bangladeshi than White, Irish, or Indian. 

### 4.2. Decomposing Income-Related Health Inequality into Its Determinants

The majority of our sample (73.8%) were women from low/middle-income countries, many of whom were likely to have migrated to South Korea through brokered international marriage, often referred to as ‘mail-order marriage’, especially during the mid-2000s. It is, therefore, not surprising that a high proportion of these women suffer from depressive symptoms in South Korea. The partial effects, however, suggested that socioeconomic positions might have stronger health implications than the type of migration itself when considering both together. The decomposition results also indicated that the type of migration had little contribution to the overall income-related health inequality among these married immigrants, but their socioeconomic status (SES), reflected in income, subjective social status, and employment status, alongside satisfaction with a spouse, made significant contributions to the overall income-related health inequality. This was even more so for self-reported depression. The contribution of demographics (age) and type of migration (the product of sex and country of origin) to the overall inequality appeared to be 23.6% for poor SRH and less than 5% for self-reported depression. The rest was mainly explained by socioeconomic positions. While the association between socioeconomic condition and health has been well-established in the literature [35,36,37], these findings suggest that socioeconomic condition could have even greater health implications, mental health in particular, among ‘*these immigrants*’ because it is often one of the most important drivers for their migration. 

This speculation is consistent with the disillusionment model, which suggests that immigrants’ mental health could deteriorate rapidly due to disillusionment and nostalgia for the past until psychological adaptation takes place [7,38,39]. The model implies that how immigrants perceive their socioeconomic position in the host country and the extent to which their post-migration reality meets their pre-migration expectation, alongside the actual level of income, are one of the key factors that determine the level of disillusionment and that of mental health. Notably, disillusionment could be even greater among those women who have migrated to South Korea through brokered international marriage because this type of migration is likely possible only when people have great expectations for the host countries. Unfortunately, however, many of these married women are likely to be located at the bottom of the socioeconomic ladder in South Korea, which is far from what they expected before migration. 

In a similar vein, the present findings highlighted the importance of married immigrants’ subjective social status (i.e., how these immigrants perceive their social status) in understanding married immigrants’ health and health inequality, particularly for self-reported depression. The prevalence of self-reported depression appeared to be lower by a 14.8 percentage point among those having high subjective social status than those having low subjective social status, independently of income and other socioeconomic factors. The equivalent for poor SRH was 4.6 percentage points. Subjective social status also accounted for 25.1% of the overall income-related inequality in self-reported depression and 10.4% in poor SRH. 

While this study did not explicitly examine the impact of the gap between pre-migration expectation and post-migration reality on health and health inequality, this could in part have been reflected in the partial effects of education, which showed no significant—but positive—association between educational attainment and self-reported depression. While this is inconsistent with the well-known effect of education on health [40], there is limited evidence that shows similar findings with immigrants and ethnic minorities [41,42,43]. For instance, Chang et al. [42] examined factors associated with health of the largest ethnic groups of married immigrant women in South Korea (Korean-Chinese, Vietnamese, and Han Chinese). The study reported that educational attainment was not very strongly associated with self-reported health, compared to subjective social status, among married immigrant women and it was even negatively associated with self-reported health among Vietnamese women in South Korea. These findings may indicate the complex mechanisms of the education effect on health among married immigrant women in South Korea. On the one hand, higher education may still have some positive effects on health among married immigrants, for instance, through healthier behaviour, but, on the other hand, it could also have some negative effects on health due to higher pre-migration expectations. That is, married immigrants with higher education could have had higher expectations for South Korea and, therefore, could have felt greater disillusionment and disappointment after migration than those with lower education. Notably, perceived discrimination could also be understood in a similar manner. Although perceived discrimination was positively associated with health outcomes, especially with self-reported depression, its distribution across income ranks appeared to be rather constant, making its contribution to the overall income-related health inequality very small. While this could mean that immigrants equally experienced discrimination regardless of their income, it is also possible that those with higher income, despite fewer objective experience of discrimination, more easily perceived discrimination than those with lower income because of their higher expectations. 

As a final note, satisfaction with a spouse appeared to have significant health implications, particularly for self-reported depression, among married immigrants in South Korea, consistent with previous findings [6]. Compared to married immigrants very dissatisfied with their spouses, the prevalence of self-reported depression and poor SRH was lower by 40.9 percentage points and 8.5 percentage points among those very satisfied with their spouses. A higher level of satisfaction (i.e., being very satisfied with a spouse) was also unequally distributed across income ranks, favouring the rich. Consequently, satisfaction with a spouse appeared to explain 31.4% of the income-related inequality in self-reported depression and 4.3% of that in poor SRH. While cultural differences and communication problems often contribute to poor marital relationship among married immigrants, existing evidence also highlights high prevalence of alcohol problems and domestic violence among the husbands of ‘mail-order brides’ in South Korea [44], which are all likely to be more prevalent among lower socioeconomic groups. 

### 4.3. Study Limitations

The present findings should, however, be interpreted with some caution due to the following study limitations. Firstly, this study used the data involving the largest number of married immigrants in South Korea, but its cross-sectional design precludes causal inference, a problem shared with almost all studies of health inequalities. The cross-sectional data, nevertheless, provide some early evidence in an area where there is currently no good source of representative health panel data for immigrants in South Korea. Secondly, this study used self-reported/self-assessed health data. While this type of data is commonly collected in large-scale surveys, self-assessment naturally involves a respondent’s subjective judgment that can vary with his or her sociodemographic background. For instance, people with higher socioeconomic status may have higher health expectations and, therefore, assess their health more negatively than those with lower socioeconomic status, even if two groups have similar levels of ‘objective’ health [45]. This issue may be more problematic for SRH, given that the assessment of depression, by definition, involves an individual’s subjective experience and judgement. Therefore, the use of poor SRH could under-estimate the true degree of income-related health inequality, although it is known to be strongly associated with morbidity and mortality [16]. Finally, this study calculated income-related health inequality based on gross income, not net income. While net income is more appropriate for health inequality studies as it takes into account the effects of income re-distribution policies, the 2015 National Survey of Multicultural Families collected only gross income. Nevertheless, this issue would be less problematic because the proportion of high-income earners is likely to be very low among immigrants. More importantly, income was collected in categories (eight categories) to reduce missing data and improve the response accuracy. The relationship between income and health within the same income categories was, therefore, not reflected in the present concentration indices. Nevertheless, these concentration indices can still show the overall relationship between income and health among married immigrants in South Korea. 

## 5. Conclusions

Despite these limitations, the present study provided evidence supporting substantial pro-rich inequalities in the prevalence of self-reported depression and poor SRH among married immigrants in South Korea. While married immigrant men from high-income countries exhibited the lowest prevalence of both self-reported depression and poor SRH than other types of immigrants, the decomposition results confirmed that demographics and type of migration had only small contributions to the overall health inequality, especially that in self-reported depression. Instead, socioeconomic factors, such as income, subjective social status, and employment status, appeared to play important roles in the overall income-related health inequality. Satisfaction with a spouse also made considerable contribution to the overall inequality in self-reported depression. Notably, however, the role of educational attainment in shaping the overall health inequality was not clear among these married migrants. These findings overall suggest that health inequalities are largely ‘avoidable and unnecessary’ among married immigrants in South Korea. This urges the need for strengthening social welfare policies and programmes for vulnerable immigrants, especially those with low SES, to improve their health. Particular attention should also be paid to mental health of low-SES women from low/middle-income countries, especially in the early phase of their migration when the gap between pre-migration expectation and post-migration reality is greatest. While this study examined the issue of health inequality only among married immigrants, further research is needed to examine the same issue across the whole population, including immigrant minorities, to better understand the socioeconomic position of these minorities in Korean society and its full health implications. 

## Figures and Tables

**Table 1:** Prevalence of self-reported depression and poor SRH by sociodemographic factors (weighted) (*n* = 15,321).

Variables	Depression	No Depression	Poor SRH	No Poor SRH
**Age, %**				
≤34	37.4	62.6	3.2	96.8
35–49	32.8	67.2	8.9	91.1
50–64	30.2	69.8	21.0	79.0
≥65	40.5	59.5	54.8	45.2
**Sex and Country of origin, %**				
Women from LMI countries	37.0	63.0	8.9	91.1
Women from HI countries	31.2	68.8	8.7	91.3
Men from LMI countries	27.1	72.9	14.5	85.5
Men from HI countries	23.0	77.0	5.0	95.0
Years of residence, mean (SD)	9.56 (6.02)	10.17 (6.88)	12.52 (7.42)	9.69 (6.45)
Rural residence, %	34.2 *	65.8 *	6.4	93.6
Urban residence, %	34.8 *	65.2 *	10.4	89.6
Disability card holder, %	55.3	44.7	53.3	46.7
Non-disability card holder, %	34.5	65.5	9.1	90.9
**Spouse satisfaction, %**				
Very dissatisfied	70.9	29.1	23.5	76.5
Dissatisfied	70.3	29.7	17.6	82.4
Fair	47.2	52.8	13.3	86.7
Satisfied	31.7	68.3	7.9	92.1
Very satisfied	22.5	77.5	6.5	93.5
Perceived discrimination, %	43.2	56.8	10.9	89.1
No perceived discrimination, %	28.8	71.2	8.5	91.5
Nobody available for help when sick, %	39.7	60.3	12.7	87.3
Somebody available for help when sick, %	31.9	68.1	7.7	92.3
**Education, %**				
≤Middle school	37.5	62.5	14.3	85.7
High school	34.7	65.3	9.1	90.9
≥University	31.5	68.5	4.9	95.1
**Employment, %**				
Regular	29.1	70.9	5.6	94.4
Temporary/day labour	36.7	63.3	10.4	89.6
Not working	37.8	62.2	12.1	87.9
**Subjective social status, %**				
Low	44.6	55.4	17.8	82.2
Middle	30.2	69.8	5.5	94.5
High	20.9	79.1	3.1	96.9

* *p* < 0.05 except for this variable; Note: The numbers indicate row percentages, except for the duration of residence. Abbreviations: HI, high-income; LMI, Low/middle-income; SD, standard deviation; SRH, self-rated health.

**Table 2:** Concentration index for self-reported depression and poor SRH.

Income Level & CI	Depression	Poor SRH
Income level, (unit = 10,000 KRW)		
<100 (*n* = 865)	49.0%	34.7%
100–200 (*n* = 3105)	40.1%	14.2%
200–300 (*n* = 5073)	35.8%	7.5%
300–400 (*n* = 3420)	31.7%	6.2%
≥400 (*n* = 2858)	25.7%	4.0%
Uncorrected CI(SE)	−0.0937 (0.0070)	−0.3247 (0.0189)
Indirectly std. uncorrected CI(SE)	−0.0923 (0.0068)	−0.2064 (0.0142)
Erreygers CI(SE)	−0.1298 (0.0097)	−0.1231 (0.0072)
Wagstaff CI(SE)	−0.1433 (0.0108)	−0.3587 (0.0209)

Note: *p*-values for all CI <0.001; Abbreviations: CI, concentration index; KRW, Korean Won; SE, standard error; SRH, self-rated health.

**Table 3:** Results of the decomposition analysis for the income-related inequality in self-reported depression and poor SRH.

Sociodemographic Factors	Exploratory Variables		Depression		Poor SRH	
	$\bar{X}$	CI	β	Cont.	β	Cont.
Duration of residence	9.960	0.004	−0.002 *	0.000	0.001 **	0.000
**Age**						
35–49	0.392	0.074	−0.048 **	−0.006	0.049 **	0.006
50–64	0.147	−0.105	−0.097 **	0.006	0.127 **	−0.008
≥65	0.027	−0.723	−0.052	0.004	0.318 **	−0.025
**Sex and Country of origin**						
Women from LMI countries	0.757	−0.023	0.024	-0.002	0.035 **	−0.002
Women from HI countries	0.058	0.160	0.020	0.001	0.038 **	0.001
Men from LMI countries	0.142	−0.044	−0.046	0.001	0.025 *	−0.001
Rural residence	0.243	−0.074	−0.020 *	0.001	−0.015 **	0.001
Disability card holder	0.007	−0.468	0.202**	−0.003	0.147**	−0.002
**Satisfaction with a spouse**						
Dissatisfied	0.042	−0.162	0.016	0.000	−0.025	0.001
Fair	0.284	−0.100	−0.184 **	0.021	−0.058 *	0.007
Satisfied	0.288	−0.007	−0.329 **	0.003	−0.083 **	0.001
Very satisfied	0.376	0.104	−0.409 **	−0.064	−0.085 **	−0.013
Perceived discrimination	0.405	0.022	0.128 **	0.004	0.028 **	0.001
No one for help when sick	0.353	−0.067	0.046 **	−0.004	0.027 **	−0.003
Education						
High school	0.436	−0.016	0.004	0.000	−0.019 **	0.001
≥University	0.272	0.175	0.018	0.003	−0.036 **	−0.007
Employment						
Temporary/day	0.304	0.026	0.041 **	0.001	0.009	0.000
Not working	0.374	−0.195	0.055 **	−0.016	0.035 **	−0.010
**Income level, (unit = 10,000 KRW)**						
100–200	0.200	−0.684	−0.075 **	0.041	−0.030 *	0.016
200–300	0.330	−0.154	−0.097 **	0.020	−0.055 **	0.011
300–400	0.231	0.407	−0.107 **	−0.040	−0.053 **	−0.020
≥400	0.181	0.819	−0.134 **	−0.080	−0.065 **	−0.039
**Subjective social status**						
Middle	0.626	0.104	−0.095 **	−0.025	−0.040 **	−0.010
High	0.041	0.322	−0.148 **	−0.008	−0.046 **	−0.002
Residual	-	-	-	0.0106	-	−0.0262
**Total CI**	**-**	**-**	**-**	**−0.1298**	**-**	**−0.1231**

* *p* < 0.05, ** *p* < 0.01, Abbreviations: CI, concentration index; SRH, self-rated health; Reference group: ≤34 years old, men from high-income countries, urban residence, no disability card holder, very dissatisfied with a spouse, no perceived discrimination, someone around for help when sick, ≤middle school, regular worker, income less than 100, low subjective social status.
